# Direct Intravascular Observation of Trifecta️ Bioprosthetic Valve Dysfunction Using Nonobstructive General Angioscopy

**DOI:** 10.1155/cric/7201873

**Published:** 2026-08-19

**Authors:** Jumpei Suzuki, Tsuyoshi Kobayashi, Toru Yoshizaki, Akira Sato

**Affiliations:** ^1^ Department of Cardiology, University of Yamanashi, Yamanashi, Japan, yamanashi.ac.jp

## Abstract

Although surgical reintervention remains the standard of care for failed surgical valves, transcatheter aortic valve implantation in surgical aortic valves (TAV‐in‐SAV) has emerged as a viable alternative with reliable results. The Trifecta valve is one of the most challenging SAVR prostheses for TAV‐in‐SAV management. Recently, nonobstructive general angioscopy (NOGA) has been used to evaluate the quality of plaques on the inner surface of the aorta under direct visualization, which cannot be evaluated using computed tomography. Here, we describe a case of Trifecta️ bioprosthetic valve dysfunction (BVD) directly observed by NOGA. A 26‐mm Evolut FX was implanted, and the procedure was completed without complications. This case highlights the potential adjunctive role of NOGA in the assessment of BVD; however, its incremental clinical benefit and safety profile require further investigation.

## 1. Introduction

There has been a trend towards bioprostheses among patients with aortic stenosis to avoid anticoagulant treatments. Consequently, the proportion of patients at risk of bioprosthetic valve dysfunction (BVD) will continue to increase in the near future. Surgical reintervention has been the standard of care; however, transcatheter aortic valve implantation for failed surgical valves (TAV‐in‐SAV) has emerged with good results [1]. Therefore, it is important to identify the cause of BVD to determine whether treatment with TAV‐in‐SAV is appropriate.

Recent studies have shown that it is now possible to observe the aortic wall lumen using a nonobstructive general angioscopy (NOGA) (Visible️, FiberTech, Japan) and that spontaneous plaque rupture occurs within the aorta, scattering cholesterol crystals to the peripheral organs [2]. Furthermore, NOGA enables direct evaluation of the quality of plaque on the inner surface of the aorta under direct visualization, which cannot be evaluated by computed tomography [3]. Here, we describe a case of Trifecta️ BVD directly observed by NOGA.

## 2. Case Presentation

**Figure 1:**
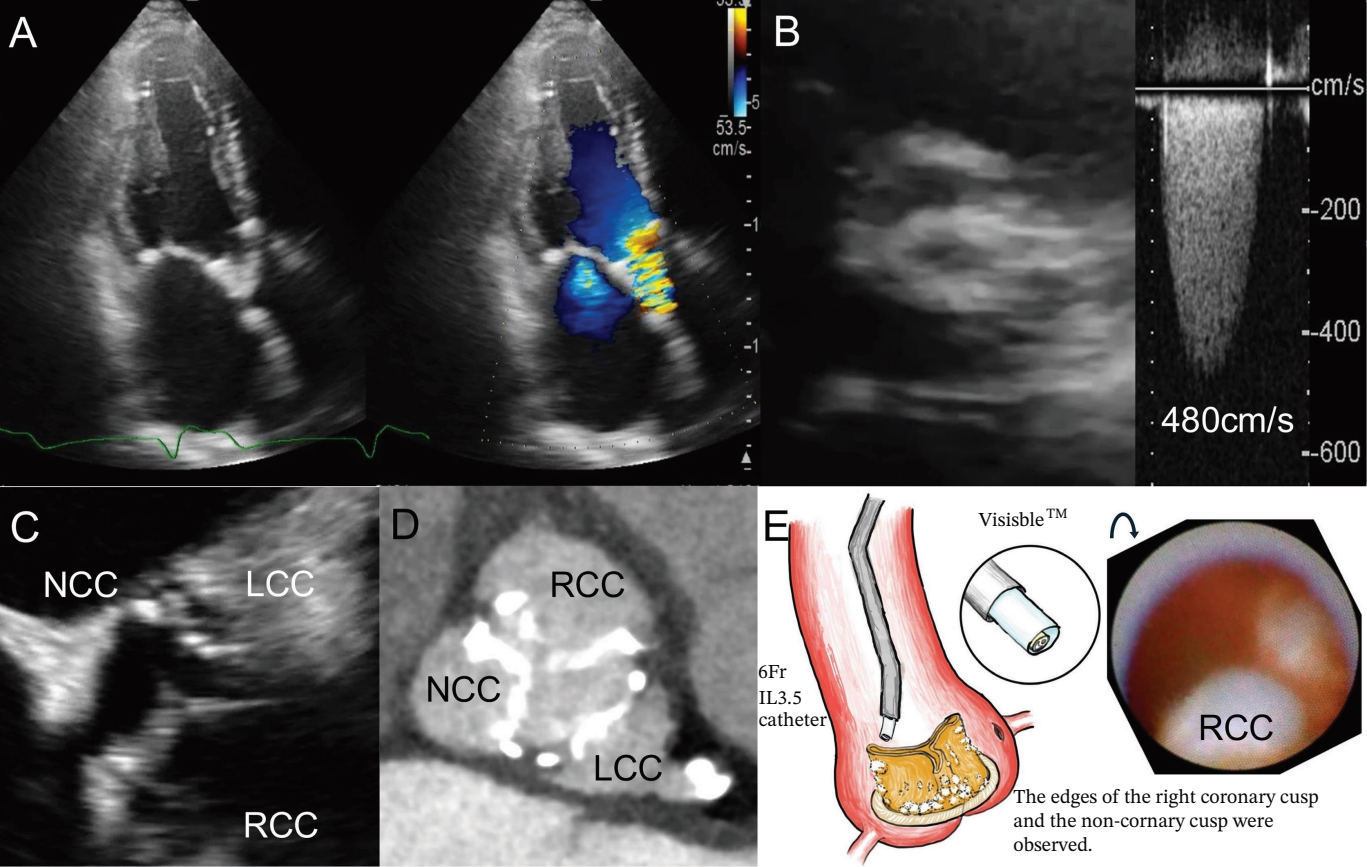
(A and B) Transthoracic echocardiography showed severe aortic stenosis, (C) transoesophageal echocardiography showing that the left coronary cusp was mobile, whereas the right and noncoronary cusps had restricted mobility, (D) computed tomography showing reduced mobility of the right and noncoronary cusps compared with the left coronary cusp in the systolic phase and (E) schematic of the angioscopy procedure.

**Figure 2:**
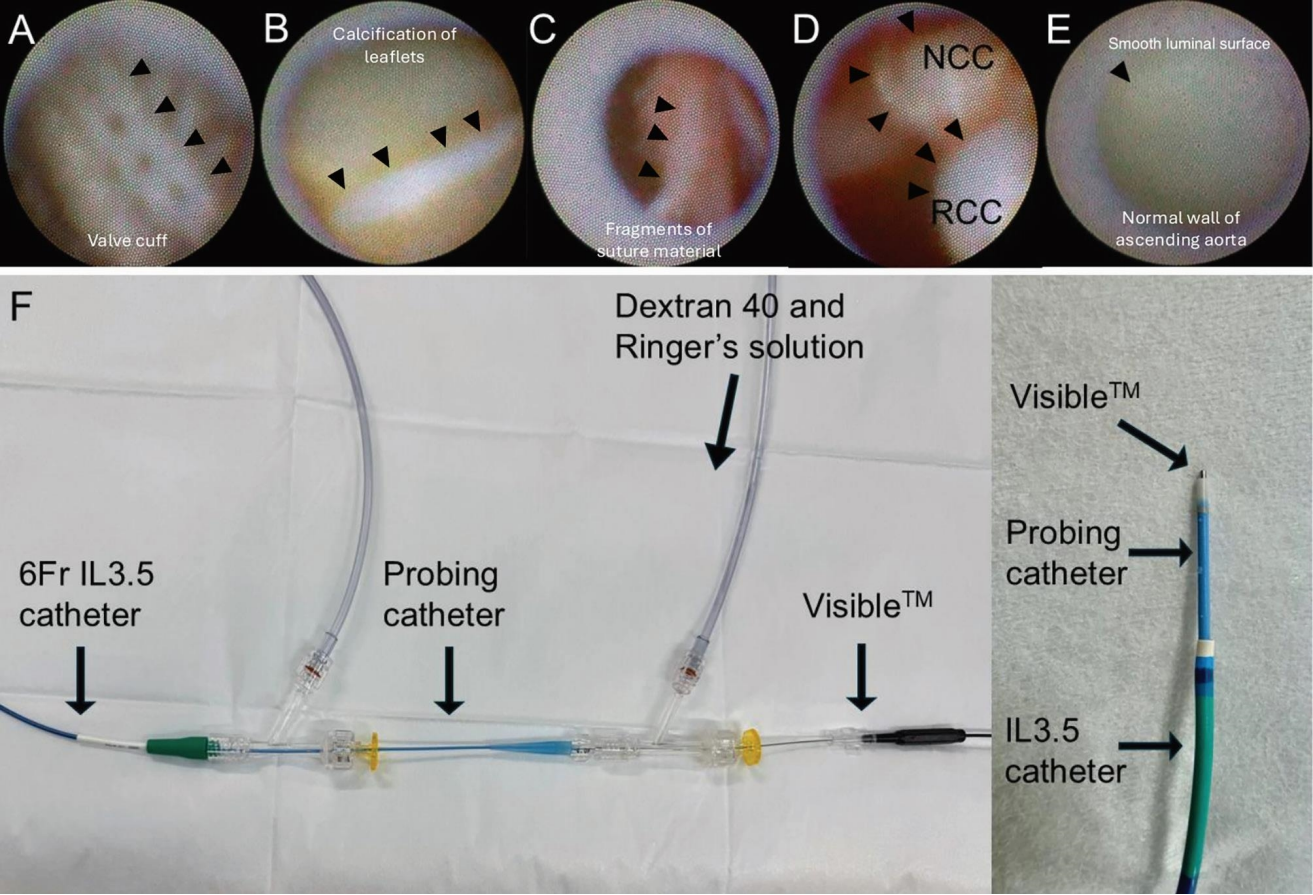
(A) Angioscopic visualization of the valve cuff of the Trifecta valve (arrowhead), (B) severe calcification of leaflets (arrowhead), (C) fragments of suture material (arrowhead), (D) severe calcification of cusps, (E) normal wall of ascending aorta and (F) the nonobstructive general angioscopy (NOGA) system.

Approximately 8 years before presentation, a 78‐year‐old man underwent aortic valve replacement with a 23‐mm Trifecta bioprosthetic valve for severe aortic stenosis (AS). The patient presented with shortness of breath. Transthoracic echocardiography (TTE) revealed severe prosthetic valve stenosis (mean transprosthetic gradient: 60 mmHg) (Figure 1A,B; Video S1). Transoesophageal echocardiography (TEE) revealed severe calcification and reduced mobility of the right and noncoronary cusp leaflets, whereas the mobility of the left coronary cusp was preserved (Figure 1C, Video S2). Multidetector computed tomography (CT) confirmed fusion of the two affected leaflets due to severe calcification (Figure 1D, Video S3). Direct intravascular observation of BVD was performed using NOGA (Figure 1E). NOGA revealed the valve cuff (Figure 2A, Video S4), severe leaflet calcification with impaired mobility (Figure 2B, Video S5), fragments of the suture material (Figure 2C, Video S6), severe calcification of the cusps (Figure 2D, Video S7) and normal wall of the ascending aorta (Figure 2E). On CT, the annular area and perimeter were 331 mm^2^ and 64.3 mm, respectively. The diameters of the right, left and noncoronary sinuses of Valsalva were 29.6, 29.6 and 31.9 mm, respectively. The left and right coronary artery heights were 6.5 and 5.5 mm, and the left and right valve‐to‐coronary distances were 4.6 and 7.0 mm, respectively. The aortic root was large, and the valve‐to‐coronary distance was secured; therefore, the risk of coronary artery occlusion was low. Given these findings, he was diagnosed with structural valve deterioration (SVD) of Trifecta️ bioprosthetic valve. A 26‐mm Evolut FX (Medtronic, Minneapolis, Minnesota, United States) was implanted, and the procedure was completed without complications.

Angioscopic examination was performed immediately after coronary angiography using a Visible inserted into a probing catheter through a 6‐French IKARI Left 3.5 guiding catheter and a console (InterTec Medicals Co. Ltd., Japan) (Figure 2F) [2]. Systemic heparinization was maintained during the procedure according to the standard protocol for diagnostic coronary angiography, and additional heparin was administered to maintain an activated clotting time (ACT) between 250 and 300 s. Before the observation, the white balance was adjusted for colour correction. Angioscopic images were recorded using a digital video recorder for offline analysis. Recordings were made during the manual injection of 10–20 mL of 10% dextran 40 and lactated Ringer′s solution (Otsuka Pharmaceutical Factory, Tokushima, Japan) to facilitate the removal of red blood cells from the observation site without ventricular overdrive pacing. The total volume of the solution used in each examination was 50–80 mL. The total procedure time for angioscopy was 10 min.

## 3. Discussion

Although surgical reintervention remains the standard of care for failed surgical valves, transcatheter aortic valve implantation in surgical aortic valve (TAV‐in‐SAV) has emerged as a viable alternative with reliable results [1]. The Trifecta valve has been reported to have limited long‐term durability [4]. In general, externally mounted leaflet valves such as the Trifecta valve are associated with a higher risk of coronary obstruction during TAV‐in‐SAV [5]. Therefore, the Trifecta valve is considered one of the most challenging surgical bioprostheses for valve‐in‐valve TAVI.

In the present case, NOGA provided direct intravascular visualization of the prosthetic valve, including the leaflet calcification, reduced mobility, valve cuff structure and remnants of the suture material. The mechanism of valve failure was considered to be most consistent with calcific SVD. Echocardiography and CT demonstrated severe calcification, fusion of the affected leaflets and restricted leaflet mobility. NOGA directly confirmed a calcified leaflet surface without apparent thrombotic material. No definite imaging findings suggested that leaflet tears or pannus were the dominant mechanisms. Direct visualization by NOGA was therefore considered clinically valuable to further characterize leaflet morphology and to assess for thrombotic changes that could influence diagnostic certainty. Nevertheless, because the valve was not surgically explanted, histopathological confirmation was not possible; therefore, the mechanism should be interpreted as an imaging‐based diagnosis.

Although these findings increased the diagnostic confidence, we acknowledge that TAV‐in‐SAV might have been selected based on conventional imaging alone. Intravascular angioscopy within the aortic root and prosthetic valve may carry potential risks including embolic complications due to plaque or debris dislodgement, mechanical trauma to the prosthetic valve and catheter‐related complications. In this patient, NOGA was performed using a 6‐Fr guiding catheter with careful manual injection of 10% dextran 40 and lactated Ringer′s solution to obtain a clear field without rapid pacing. No procedural complications, haemodynamic instability, embolic events or valve injuries were observed. Cerebral embolic protection was not used in this case; however, it may be considered in selected future cases, particularly in patients with a heavy aortic plaque burden or high embolic risk. Because systematic data regarding NOGA in the aortic root and prosthetic valves are limited, careful patient selection and procedural expertise are essential.

Furthermore, while NOGA provides superior surface‐level spatial resolution and direct visualization compared with CT and echocardiography, it cannot replace CT for procedural planning, particularly for evaluating anatomical feasibility, coronary height and valve‐to‐coronary distance (Table 1). Thus, NOGA should be considered a complementary tool rather than a standalone modality. In selected cases where the mechanism of BVD remains uncertain, NOGA may provide additional morphological information. Furthermore, as leaflet modification techniques, such as BASILICA [6], UNICORN and the ShortCut device, are increasingly used to reduce the risk of coronary obstruction, NOGA may have potential value for real‐time leaflet assessment or procedural support. Currently, NOGA is performed under two‐dimensional fluoroscopic guidance. In the future, the incorporation of CT image fusion or 3D mapping systems—similar to those used in catheter ablation—could significantly improve three‐dimensional spatial orientation and enable more precise correlation between angioscopic and conventional imaging findings. Their application remains speculative and requires further investigation.

**Table 1:** Potential complementary roles of echocardiography, CT and NOGA in the evaluation of bioprosthetic valve dysfunction.

Modality	Strengths	Limitations	Potential clinical role
Echocardiography (TTE/TEE)	Haemodynamic assessment; transprosthetic gradient; regurgitation; leaflet motion; bedside availability	Limited tissue characterization; acoustic shadowing; incomplete assessment of leaflet surface pathology	First‐line diagnosis and severity grading; follow‐up of prosthetic valve function
CT	Anatomical assessment; leaflet calcification or thickening; valve sizing; coronary obstruction risk assessment	Radiation and contrast exposure; indirect tissue characterization; limited temporal resolution for leaflet motion	Mechanism assessment and procedural planning for valve‐in‐valve TAVI
NOGA	Direct real‐time visualization of leaflet surface and motion under blood displacement	Invasive; limited field of view; requires interpretive expertise; potential embolic or traumatic risk; limited safety data in prosthetic valves	Adjunctive assessment in selected cases when the mechanism remains uncertain; possible support for procedural planning

## 4. Conclusion

Determining the mechanisms underlying BVD is essential for optimal management. In this case, NOGA provided direct visualization of prosthetic valve degeneration, enabling a detailed assessment of leaflet morphology and supporting the diagnosis of structural valve deterioration. NOGA may serve as a complementary imaging modality in selected cases. It may provide additional morphological information when conventional imaging findings are inconclusive regarding the mechanism of BVD. However, further studies are required to clarify its clinical utility and safety in this setting.

## Author Contributions

J.S. and T.K. contributed to the conception and design of this study and drafting of the article. T.Y. and A.S. contributed to critical revision of the manuscript for important intellectual content.

## Funding

No funding was received for this manuscript.

## Consent

Written informed consent was obtained from the patient for publication of this case report and accompanying images.

## Conflicts of Interest

The authors declare no conflicts of interest.

## Supporting Information

Additional supporting information can be found online in the Supporting Information section.

## Supporting information


**Supporting Information 1.** Supplementary videos demonstrating multimodality imaging and nonobstructive general angioscopic findings of the Trifecta bioprosthetic valve dysfunction. Video S1: Transthoracic echocardiography demonstrates severe prosthetic aortic stenosis.

**Supporting Information 2.** Video S2: Transoesophageal echocardiography shows severe calcification and restricted motion of the right and non‐coronary cusps, whereas the left coronary cusp remained mobile.

**Supporting Information 3.** Video S3: Multidetector computed tomography shows reduced mobility of the right and non‐coronary cusps compared with the left coronary cusp in the systolic phase.

**Supporting Information 4.** Video S4: Nonobstructive general angioscopy (NOGA) shows the valve cuff structure of a bioprosthetic valve.

**Supporting Information 5.** Video S5: NOGA shows severely calcified bioprosthetic valve leaflets. The whitish leaflet surfaces corresponded to calcific degeneration. No evident thrombotic material was observed.

**Supporting Information 6.** Video S6: NOGA shows remnants of suture material attached to the prosthetic valve.

**Supporting Information 7.** Video S7. NOGA shows marked calcification of the cusps and markedly restricted leaflet motion during the cardiac cycle.

## Data Availability

Additional data used to support the findings of this study are available from the corresponding author upon request.
